# Ovarian Cancer Screening Practices of Obstetricians and Gynecologists in Puerto Rico

**DOI:** 10.1155/2014/920915

**Published:** 2014-06-05

**Authors:** Gianni Rodríguez-Ayala, Josefina Romaguera, Mariel López, Ana P. Ortiz

**Affiliations:** ^1^Department of Obstetrics and Gynecology, University of Puerto Rico School of Medicine, San Juan, PR 00936-5067, USA; ^2^Department of Biostatistics and Epidemiology, University of Puerto Rico School of Medicine, San Juan, PR 00936-5067, USA; ^3^Cancer Control and Population Sciences Program, University of Puerto Rico Comprehensive Cancer Center, San Juan, PR 00927-6346, USA

## Abstract

*Background.* Ovarian cancer is the most fatal malignancy of the female genital tract and is associated with high mortality. The American Congress of Obstetricians and Gynecologists (ACOG) and the United States Preventive Services Task Force (USPSTF) recommend against screening for ovarian cancer in asymptomatic, average-risk women. *Objective.* To assess the ovarian cancer screening practices in asymptomatic, average-risk women among obstetricians and gynecologists (Ob/Gyn) in Puerto Rico. *Methodology.* From 2011 to 2012, self-administered anonymous questionnaires were mailed to all licensed obstetricians and gynecologists in PR. *Results.* Response rate was 25%. Overall, 53.9% were screening for the disease. Reported screening methods were CA-125 and transvaginal ultrasound (TVUS), 39.2%, TVUS only, 30.4%, and CA-125 only, 9.8%. In the logistic regression model, the odds that a given health practitioner routinely screened for ovarian cancer in the asymptomatic, average-risk population increased by 8% with every unit increase in his or her years in practice. *Conclusion.* The majority of the practicing Ob/Gyn in PR who participated are not following the guidelines established by the ACOG and the USPSTF for ovarian cancer screening.

## 1. Introduction

Ovarian cancer is a low-incidence but highly lethal disease, which makes it the most fatal malignancy of the female genital tract. It is the fifth and seventh leading cause of death among women in the United States (US) and Puerto Rico (PR), respectively [1, 2]. In PR, ovarian cancer accounted for 2.5% of all female cancers from 2006 through 2010 and 4.2% of all female cancer-related deaths from 2006 through 2010. Every year, approximately 153 new cases are diagnosed in PR [2]. Ovarian cancer tends to be diagnosed when it has already reached an advanced, often lethal, stage. This tendency toward late diagnosis is most likely caused both by the lack of an effective screening method and by the absence of early symptoms [3, 4]. By the time of diagnosis, most patients have already reached stage III (58%) or even stage IV (17%) of the disease [5, 6]. For women at an advanced stage, the 5-year survival rate is 28 to 45%, while those diagnosed early have a 95% survival rate [7, 8]. This marked difference in survival rates makes the need for reliable screening tests obvious. Despite this, the American Congress of Obstetricians and Gynecologists (ACOG), the Society of Gynecologic Oncologists (SGO), and the U.S. Preventive Services Task Force (USPSTF) currently recommend against screening for ovarian cancer, for the reasons detailed in Table 1 [9–11].

Numerous efforts have been directed towards the identification of a reliable screening test. The Prostate, Lung, Colorectal and Ovarian (PLCO) Cancer Screening Trial [7] was a large, prospective study conducted in the United States. The objective of this trial was to determine whether CA-125 plus transvaginal ultrasound (TVUS) could reduce mortality from ovarian cancer in asymptomatic women aged between 55 and 74 years. In the baseline screening round, 28,732 women had their CA-125 levels measured and 28,478 underwent TVUS. These tests were found to be abnormal in 1.4% and 4.6% of the test subjects, respectively, and only 0.1% of the subjects had abnormalities in both tests. The positive predictive value of these tests for invasive cancer was 3.7% for an abnormal CA-125 test, 1% for an abnormal TVUS, and 23.5% if both tests were abnormal. The study by Pavlik and Van Nagell [12] presents a review of 4 of the major studies on ovarian cancer screening methods that are going on at this time, including the PLCO Trial [7, 13], the University of Kentucky Ovarian Cancer Screening Trial [14], the United Kingdom Collaborative Trial of Ovarian Cancer Screening (UKCTOCS) [15], and the Shizuoka Cohort Study on Ovarian Cancer Screening (SCSOCS) Trial [16]. They conclude that ovarian cancer screening is still in the early phases of development.

Based on the available data and current guidelines discussed above, obstetricians and gynecologists should not be routinely screening the asymptomatic, average-risk women for ovarian cancer. Given the lack of data on the ovarian cancer screening practices of physicians in Puerto Rico, our objective in this study was to assess such practices of obstetricians and gynecologists in this average-risk population. This information would be valuable in the implementation of more aggressive patient and physician education programs since undergoing nonvalid screening tests could result in increased costs, increased patient anxiety, and unnecessary surgeries.

## 2. Methodology

After obtaining Institutional Review Board approval from the Medical Sciences Campus of the University of Puerto Rico, a self-administered anonymous questionnaire was mailed to all of the licensed obstetricians and gynecologists (*n* = 440) in Puerto Rico from 2011 through 2012. Participants were not asked to specify if they had any subspecialty. We believe this had no effect on selection bias, as there were only 3 gynecologic oncologists in the whole island during the study period. Participants were followed up by sending 2 mail letters. Addresses were obtained from the corresponding section of the College of Physicians and Surgeons of Puerto Rico. Nonpracticing physicians were excluded from the study. The questionnaire included general questions related to age, gender, practice setting, and years in practice as well as specific questions on the use of screening tests for ovarian cancer in the asymptomatic, average-risk population.

### 2.1. Definition of Study Variables

The ages of the study participants (range: 31–85 years) were described as a continuous variable. Practice setting was defined as* private*,* government*,* academic*, or* combined*. The following dichotomous variables (yes/no) were also included in the questionnaire: (1) whether the physician normally recommended routine screening for ovarian cancer and (2) three different variables about the modalities that physician most commonly used as a first test to screen for ovarian cancer: (a) CA-125, (b) transvaginal ultrasound, and (c) both. The ages of the patients when their physicians began and stopped screening them for ovarian cancer were collected as a continuous variable. The frequency of screening for ovarian cancer in asymptomatic, average-risk women was analyzed as a categorical variable. This variable was classified into the following 9 categories:* yearly*,* every 2 years*,* every 3 years*,* every 1 to 2 years*,* every 2 to 3 years*,* every 3 to 5 years*,* at every visit*,* according to symptoms*, and* occasionally*. The reasons provided in questionnaires by participants for not screening for ovarian cancer were categorized as (1)* too expensive*, (2)* unproved effectiveness*, or (3)* other (responses included high cost, unproved effectiveness or were left blank)*. Information regarding different sources of knowledge for current screening recommendations was also collected; those sources were (1)* professional organization statements*, (2)* medical journals*, (3)* scientific meetings*, and (4)* other*.

### 2.2. Statistical Analysis

Normally distributed data was summarized as means with their respective standard deviations, and non-normally distributed data was presented as medians with their respective percentiles (P_25_, P_75_). Categorical data was summarized as frequency distributions. Comparisons of proportions and means between ovarian cancer screening practices (yes/no) groups were based on Fisher's exact/Pearson's chi-squared test and the *t*-test, respectively. For dependent variables not normally distributed, the Wilcoxon-Mann-Whitney test was used. Logistic regression modeling was used to determine the factors associated with ovarian cancer screening practices. For all tests, a *P* value of less than 0.05 was considered statistically significant. Statistical analysis was performed using STATA, v. 11.2.

## 3. Results

### 3.1. Characteristics of the Participating Physicians

Questionnaires were returned by 102 participants (25% response rate).  Table 2 shows physician characteristics, including mean age, which was 55.1 ± 11.1 years. The mean age of obstetricians and gynecologists in ACOG District IV (which includes Puerto Rico) is 51 years and national average age is 50.7 years. At present we do not have a reproducible database available in Puerto Rico to obtain physicians' demographics [17]. A total of 74 male physicians (72.5%) and 22 female physicians (21.6%) completed the questionnaire; 6 (5.9%) were left blank. Half of the participating physicians (50%) fell in the range of 41 to 60 years. The mean years in practice were 24.4 ± 11.1. The practice setting was distributed as follows: (1) private, 79%; (2) academic, 3%; and (3) combined, 18%. Of the 17 physicians that reported having a practice in more than 1 setting, 12 (70.6%) had practiced in a private combined with either a government or an academic setting. More than half (52.0%) of the physicians specified the metropolitan area as being the location of their practices.

### 3.2. Screening Practices

**Figure 1:**
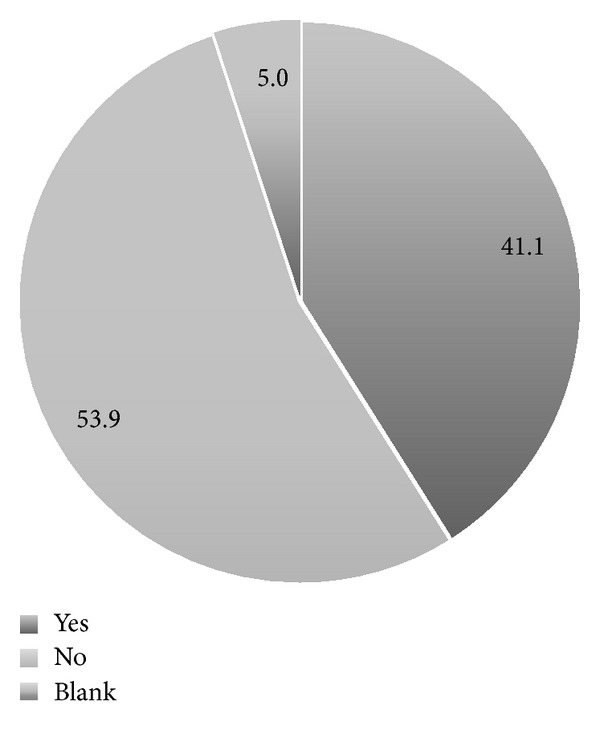
Distribution (%) of physicians' answers regarding whether or not they screen their patients for ovarian cancer (*n* = 102).

**Figure 2:**
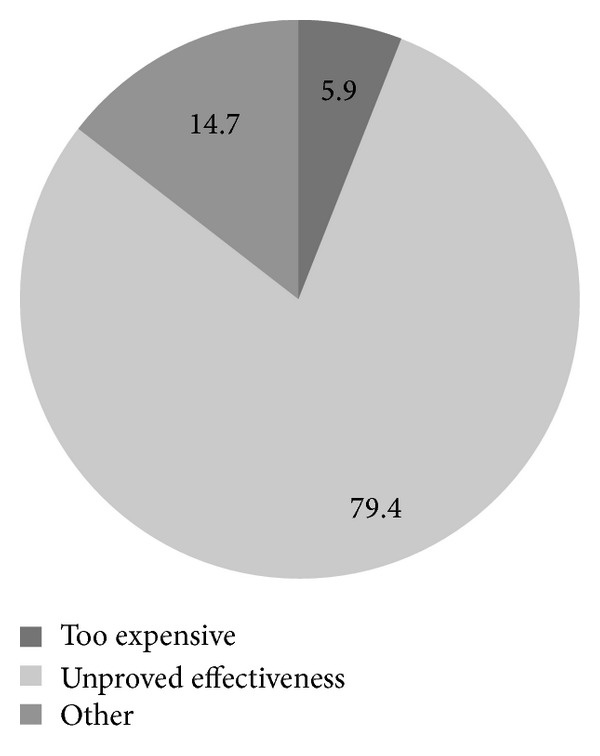
Distribution (%) of physicians' acknowledged reasons for not recommending ovarian cancer screening to their patients (*n* = 34).

**Figure 3:**
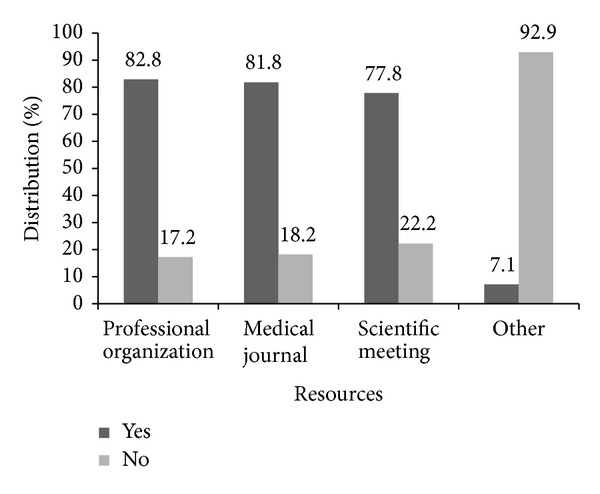
Distribution (%) of the resources used by the participating physicians to learn about current screening recommendations for ovarian cancer (*n* = 99).

Approximately half of the physicians (53.9%) routinely screened their asymptomatic, average-risk patients for ovarian cancer despite their low risk for the disease and no genetic or family history (Figure 1). The distribution of modalities used as a first test to screen for ovarian cancer was as follows: (1) transvaginal ultrasound, 31.3%; (2) CA-125, 10.2%; and (3) both, 40.4%. Twenty-eight percent of the physicians did not perform any screening in the initial visit. The mean patient age at which physicians start screening was 41.7±9.2 years. Most of the physicians never stop (48.0%) screening for ovarian cancer. Fifty-nine of the physicians reported screening for ovarian cancer in average-risk women every year. Most of the physicians (79.4%) reported unproved effectiveness as a reason for not screening with either CA-125 or transvaginal ultrasound (Figure 2). Finally, the completed questionnaires included valuable information in terms of how physicians learned about current screening recommendations (*n* = 102). Most of the physicians reported professional organizations and medical journals as being their principal sources of knowledge for current screening recommendations (Figure 3).

### 3.3. Bivariate Analysis

There was a statistically significant difference between the mean age of the physicians that routinely screen their patients for ovarian cancer and that of those that do not (*P* < 0.001). The mean number of years in practice was also significantly higher (*P* < 0.001) among physicians who routinely screen for ovarian cancer (27.9 ± 1.5 years) than it was among those who do not (19.9 ± 1.6 years) routinely screen for ovarian cancer. No significant differences (*P* > 0.05) in screening practices were seen in terms of the participating physicians' genders (Table 2) or their practice setting (*P* > 0.05) (data not shown).

### 3.4. Multivariate Analysis

In the logistic regression model, the odds of routinely screening for ovarian cancer increased by 8% with every unit increase in the participating physician's years in practice (OR = 1.08; 95% CI = 1.03–1.13), after adjusting for gender (data not shown). Only years in practice were considered in the model, as this variable was strongly correlated to physician age. The remaining variables that were analyzed were not statistically significant (data not shown).

## 4. Discussion

To date, no screening test for ovarian cancer in asymptomatic, average-risk women has been recommended by any organization, including the ACOG and the USPSTF [9]. This is because of the absence of a test with the sensitivity, specificity, and positive predictive value required for a reliable screening test for the general population [18]. Evidence of this is the fact that it is well known that CA-125 levels increase in the blood serum of patients with ovarian cancer, specifically epithelial-type ovarian cancer. Since estrogen, other hormones, smoking history, obesity, age, race/ethnicity, and having had a hysterectomy also affect its level, it is not specific enough to be a reliable biomarker for this disease as using it might lead to many false-positive test results [19, 20].

To our knowledge, this is the first study to describe the use of ovarian cancer screening tests in asymptomatic, average-risk women by obstetricians and gynecologists in PR and Latin America. Our findings indicate that the majority (53.9%) of obstetricians and gynecologists in PR who participated in this survey perform ovarian cancer screening on their patients. This is in spite of the absence of recommendations supporting this practice and, in fact, the numerous studies showing evidence against it, evidence that includes the current guidelines from the ACOG and the USPSTF [10, 11].

The questionnaires used to gather the data included a section that allowed us to learn how obstetricians and gynecologists stay current on new guidelines and scientific developments. Based on the results, we have ascertained that respondents use resources such as professional organizations, medical journals, and scientific meetings to learn about current recommendations and do so more or less equally. A discrepancy is noted with the results presented above, as many of them affirmed that they recommended yearly screening for ovarian cancer using CA-125 and TVUS. This information is valuable in the implementation of more effective physician education programs at our community. It is well known that unnecessary procedures performed on patients may cause psychological harm, may affect health-related quality of life (HRQoL) and health insurance, could result in excessive personal costs, and could lead to more invasive procedures [21, 22].

Various studies have been conducted to evaluate the impact that screening for diseases, without performing any interventions, has on patients. In addition, multiple studies [21–24] have shown that reactions to abnormal screen results and diagnostic work-ups are mostly emotional and frequently include anxiety and distress. Health distress and fear of cancer and death were identified as having the greatest adverse effects on HRQoL. Even though screening programs may have a positive impact on a given patient's mortality, physicians need to be aware of the psychological impact screening results have on patients and should refer them to a psychiatric consultant, if needed. Furthermore, concern about nonadherence to guidelines has increased, even though it has been well established that adherence is mandatory in order to prevent unnecessary testing that can cause harm to patients. Another investigator reported results similar to our findings showing that 28% of physicians reported nonadherence to screening recommendations of women at low risk for ovarian cancer [25].

This study had the following limitations: a low response rate (25%), which may alter the results (selection bias) and limit that of being generalized to the entire population of obstetricians and gynecologists in PR. Nonetheless, our response rate of 25% was expected and is comparable to other mailed surveys in the US [26, 27], where, in fact, it has been recommended that incentives be used to increase said rates [28]. In addition, other specialists (e.g., family physicians) who also are involved in preventive medicine were not included in the study. Despite these limitations, our study shows that there is lack of adherence to ovarian cancer screening guidelines among obstetricians and gynecologists in Puerto Rico and thus that there is a need for better disseminating the evidence-based recommendations regarding ovarian cancer screening among this group of health care professionals. Effective educational programs directed towards physicians and patients should be implemented. Because adherence to the current guidelines has been shown to have a positive impact on patients, future research should be aimed at discovering new tools and strategies that health care workers can use to emphasize the importance of such adherence.

## Figures and Tables

**Table 1:** Current guidelines as established by the ACOG and the USPSTF.

ACOG and SGO [10]	USPSTF [11]
The ACOG and the SGO believe that currently there are no effective screening strategies for routine ovarian cancer screening in the asymptomatic average-risk patients. They do recommend an annual gynecologic examination with an annual pelvic examination for preventive health care in this population.	The USPSTF recommends against routine screening for ovarian cancer. *See recommendation D* ^a^ * below*.

^a^ *U.S. Preventive Services Task Force Recommendations and Ratings*. The Task Force grades its recommendations according to 1 of 5 classifications (A, B, C, D, and I) reflecting the strength of evidence and magnitude of net benefit (benefits minus harms). In recommendation D, the USPSTF advises health care workers against routinely providing ovarian screening to asymptomatic patients; the Task Force found at least fair evidence that such screening is ineffective or that it may, in fact, do more harm than good.

**Table 2:** Characteristics of participating physicians (*n* = 102).

Characteristics	Overall sample	Physicians who perform ovarian cancer screening on average-risk patients (*n* = 55)	Physicians who do not perform ovarian cancer screening on average-risk patients (*n* = 42)	*P* value
Sex				
Male	74 (72.5%)	42 (77.8%)	32 (76.1%)	0.85
Female	22 (21.6%)	12 (22.2%)	10 (23.8%)	0.85
Blank	6 (5.9%)			
Age in years (mean ± SD)	55.1 ± 11.1	58.6 ± 1.5	50.6 ± 1.6	<0.001
Years in practice (mean ± SD)	24.4 ± 11.1	27.9 ± 1.5	19.9 ± 1.6	0.003
